# Penetrating Wounds of the Eye

**Published:** 1899-10

**Authors:** A. Bethune Patterson

**Affiliations:** Atlanta, Ga.


### PENETRATING WOUNDS OF THE EYE.

BY A BETHUNE PATTERSON, M.D.,
ATLANTA, GA.

The family physician is usually the first to see patients with penetrating wounds of the eye, and as so much hinges upon the advice given as to the course to be pursued in the management of such grave cases, the rules laid down for guiding the eye surgeon should be better understood. I have known a number of serious results to follow what I conceived to be a lack of appreciation of the gravity of such injuries.

Penetrating wounds of the eyeball, and markedly so in the ciliary region, are the most serious lesions we are called upon to treat, not only from the almost certainty of causing total blindness, but the frequency with which such injuries lead to sympathetic ophthalmia and blindness in the fellow eye.

The ciliary region is often spoken of as the "dangerous zone," because of the fact that penetrating wounds in this region are so often followed by iridocyclitis. Such injuries at the onset do not always present symptoms which are distressing or alarming to the patient. Sight may not be greatly impaired, which to the unprofessional is often misleading; they reason as long as there is any sight there is no cause for alarm, and a physician's services are considered unnecessary.

It is no easy matter to impress patient and friends with the gravity of such injuries and the importance of prompt action in dealing with such accidents. When they have been informed of the danger of sympathetic ophthalmia and the importance of immediate enucleation, they start in search of conservative doctors, and feel comforted when advised to wait for further developments.

Advice in a matter of this kind should not be based upon personal experience solely, but upon the accumulative experience of eye surgeons of long observation.

Modern etiology and pathology point strongly to infection as the source of danger in sympathetic ophthalmia. The word sympathetic is, to say the least, misleading. A very small wound opens the way to the introduction of germs of an inflammatory nature. The ciliary region offers to such germs suitable soil for propagation. The pathological germs find their way into the sympathizing eye either by way of the blood or lymph channels, and an inflammation is set up which is obstinate and destructive, rarely terminating otherwise than in total blindness, and often destruction of the eyeball. I am inclined to the view that the reason why the tissues of the eye are more susceptible than other tissues of the body to attacks of sympathetic inflammation, is due to a peculiar nervous connection which appears to exist from eye to eye. The conditions which are quickly established in an eye after its fellow has been injured is a commonplace experience. The eye waters and becomes intolerant to light. The blood-vessels become congested, and the eye altogether has an inflamed appearance. The tissues thus affected offer little resistance to the invasion by germs of an inflammatory character.

Sympathetic irritation and sympathetic inflammation are separate conditions; the former is nervous and functional, precedes the latter, and becomes copartner. Some of the symptoms are identical, as photophobia, congestion, pain in eye and temples; sympathetic irritation never leads to pathological changes in the tissues. But in sympathetic inflammation such changes take place in the iris, choroid, as well as in the lens and vitreous. The symptoms characteristic are not always marked or very pronounced. It is often the case that little inconvenience is experienced until marked destructive changes have taken place. On the other hand, the inflammation may present itself unmasked; pain, congestion, lacrimation and photophobia may be very pronounced, with tenderness on pressure in the ciliary zone. The characteristic evidences of iritis are present—viz.: contraction of the pupil, discoloration and effusion, which frequently become organized, blocking up the pupil.

An eye once attacked with iridochoroiditis frequently becomes the subject of periodical outbreaks of pain and inflammation; the walls become thin and the eyeball becomes distended and enlarged, and often terminates in a hard, contracted organ. This chronic ophthalmia frequently goes on for years; the lens becomes opaque, the ciliary muscles and tunics degenerate, often ulcerate and collapse, terminating in a shrunken, hard mass of diseased tissue.

When is enucleation to be advised? There are cases that occasionally present themselves that will not admit of an offhand division. This is true in those cases where there is some sight in the injured eye and an uncertainty as to whether or not the symptoms in the fellow eye are those of irritation or inflammation. If the symptoms are those of iridocyclitis, enucleating the exciting eye would not be recommended, because, as has been stated, the tendency to rapid and total blindness in the sympathizing eye and the retention for an indefinite period of partial sight in the exciting or injured eye. We can with positiveness advise and urge enucleation in an eye which has just received a wound penetrating the ciliary zone, irrespective of the amount of vision remaining. I observed a unanimity among eye surgeons upon this point, not only in this country but those in London and on the continent. The same advice would hold good in an eye containing a foreign body, whether or not the dangerous zone be implicated, unless the foreign body be in the anterior chamber, in which position efforts should be made to extract it; should this fail, excision would be the proper thing to do.

Blind and staphylomatous eyes, owing to their tendency to ulcerate and collapse, as well as those which have become shrunken, hard, and tender on palpation, are safe for enucleation. Such eyes are a source of danger to the welfare of the fellow eye.

*507 English-American Building.*

